# COVID-19 prevention and preparedness among healthcare workers in Sierra Leone

**DOI:** 10.4102/jphia.v16i1.739

**Published:** 2025-04-16

**Authors:** Ifeolu David, Tyler W. Myroniuk, Mansoo Yu, Enid Schatz

**Affiliations:** 1 Department of Epidemiology, School of Public Health, University of Michigan, Ann Arbor, MI, United States of America; 2 Department of Public Health, College of Health Sciences, University of Missouri, Columbia, MO, United States of America; 3 Department of Social Work, College of Health Sciences, University of Missouri, Columbia, MO, United States of America; 4 Department of Women’s and Gender Studies, University of Missouri, Columbia, MO, United States of America; 5 Graduate School, University of Missouri, Columbia, MO, United States of America

**Keywords:** COVID-19, prevention, in-depth interviews, healthcare workers, health behaviour

## Abstract

**Background:**

Sierra Leone’s health system has faced significant challenges, including the long-term impacts of the 2014 Ebola outbreak, prolonged conflicts before that, and economic factors contributing to the fragility of healthcare systems in many low-income settings. This qualitative study explores COVID-19 prevention practices among healthcare workers in the context of their past experiences with disease outbreaks.

**Aim:**

This study aims to understand COVID-19 prevention practices among healthcare workers in Sierra Leone and how their past experiences with disease outbreaks influence these practices.

**Setting:**

The study was conducted in three districts of Sierra Leone – Freetown, Makeni and Kenema – focusing on healthcare workers in a low-income setting with ongoing public health challenges.

**Methods:**

In-depth interviews were conducted with 24 healthcare workers, and the data were analysed for themes using the Health Belief Model and Theory of Planned Behaviour.

**Results:**

Healthcare workers demonstrated positive attitudes and strict adherence to infection prevention measures, influenced by their Ebola outbreak experience. Barriers included limited personal protective equipment and social disapproval.

**Conclusion:**

Interventions should focus on improving access to infection prevention tools and combating disapproval through community engagement. These findings are crucial for enhancing infectious disease prevention among healthcare workers in low-income settings.

**Contribution:**

This study provides insights into how past outbreak experiences influence disease prevention practices among healthcare workers in Sierra Leone, highlighting the need to address adherence barriers. These findings contribute to a broader understanding of infectious disease prevention in low-income settings and enhance global efforts in preparing for future public health emergencies.

## Introduction

The World Health Organization (WHO) declared coronavirus disease 2019 (COVID-19) a public health emergency on 30 January 2020 and a pandemic on 11 March 2020.^1^ Within a year, over 700 million cases were reported globally, with the pandemic influencing all aspects of life.^2^ Healthcare workers, including doctors, nurses and other frontline staff, were particularly impacted by the pandemic^3,4,5^ because of their close proximity to infected patients.

The rapid transmission of the virus saw an increase in the burden on health systems, globally, and many facilities had to deal with shortages in capacity, staff or equipment.^6^ Between 10% and 20% of COVID-19, cases in the United Kingdom (UK) and United States (US) were estimated to be healthcare workers, for instance.^4^ Higher rates of infection among healthcare workers could be explained by their increased risk of exposure from patients, limited knowledge in dealing with the disease during the early stages of the outbreak, limited availability of personal protective equipment (PPE) and difficulties associated with early suspect case isolation and confirmed case management.^7,8^

In Sierra Leone, the West African country where this study takes place, healthcare workers – like those throughout the world – were quickly overwhelmed when the COVID-19 outbreak hit the region; the risk of exposure via potentially infected patients remained high through multiple COVID-19 surge waves experienced during the pandemic.^9^ However, there is a limited understanding of COVID-19 prevention practices among healthcare workers in sub-Saharan Africa, including Sierra Leone. Uniquely, many healthcare workers in Sierra Leone had also faced the deadly 2014 Ebola outbreak. By examining the attitudes and practices towards preventing COVID-19 among Sierra Leonean healthcare workers, the public health community can gain insight into how experiencing two major outbreaks within a short period can shape health behaviour, especially in a context where healthcare workers have comparatively far fewer resources than their high-income country counterparts.

Because there were no specific treatments for COVID-19 at the onset of the pandemic, social distancing, masking and policy interventions were implemented to slow down the spread of the COVID-19 virus in Sierra Leone.^10^ Initially, the Sierra Leonean government emphasised and enforced guidelines pertaining to social distancing, handwashing and mask wearing.^10^ In addition to rigorous contact tracing measures,^11,12^ educational institutions were shuttered (from 24 March 2020 to 01 April 2021), and religious gatherings were banned (from 24 March 2020 to 13 August 2021). Air travel was also suspended between 22 March 2020 and 22 July 2020.^10^ National lockdowns were instituted during two distinct periods, from 05 April to 07 April 2020 and from 03 May 2020 to 05 May 2020.^10^ Meanwhile, the movement of individuals between districts was prohibited from 14 April 2020 to 04 July 2020.^10,13^ Health authorities undertook a continuous process of evaluation and refinement of COVID-19 prevention policies and measures. By the end of 2022, a return to activities at levels commensurate with those observed prior to the pandemic was achieved.

Ultimately, effective prevention practices among healthcare workers were essential to controlling the spread of COVID-19 within healthcare facilities. These measures were crucial in protecting the health and safety of both healthcare workers and patients. Constructs of the Health Belief Model (HBM) and the Theory of Planned Behaviour (TPB)^14^ inform us in our sensemaking of how and why Sierra Leonean healthcare workers engaged in prevention, based on their motivations and experiences. The HBM postulates that individual health behaviour change is determined by perceptions of susceptibility, severity, benefits and barriers alongside self-efficacy and cues to actions.^15,16,17^ The TPB posits that attitudes, subjective norms and perceived behavioural control also influence behavioural intentions and health behaviour.^18,19,20,21^ Though neither the HBM or TPB is comprehensive for all contexts, they can be effective collectively^14,22^ and therefore guide our analytic approach to understanding the complex interplay of factors that drive healthcare workers’ engagement in preventive behaviours in Sierra Leone. This study aims to provide insights into COVID-19 prevention practices among healthcare workers in Sierra Leone, focusing on factors influencing their actions and informing strategies for future infectious disease preparedness.

## Research methods and design

### Research design and setting

The data used in the paper were part of a broader qualitative project exploring COVID-19 experiences, prevention behaviour and vaccine uptake among healthcare workers in Sierra Leone. In this article, ‘healthcare worker’ specifically refers to individuals employed by hospitals or public health agencies who are directly involved in patient care or public health community outreach. This includes clinical staff such as doctors and nurses, as well as public health personnel working in outreach roles, but excludes administrative and purely support roles that do not involve direct patient or public health engagement. The study was conducted in post-conflict Sierra Leone, where the healthcare system was significantly weakened by a decade-long civil war that concluded in 2001. Since then, the healthcare system has received substantial investment to support its recovery.^23^ The healthcare infrastructure in Sierra Leone also experienced significant challenges because of the Ebola outbreak in 2014, resulting in a substantial number of fatalities, including healthcare workers.^24^ As a result, the study participants had firsthand experience with the challenges posed by infectious diseases, either in their professional capacity as clinicians or within their communities, prior to their recent decision to pursue a career in healthcare.

### Participant recruitment

The sample consisted of 24 healthcare workers from three of the 16 districts in Sierra Leone: Freetown, Makeni and Kenema. These regions were particularly hard hit during the 2014 Ebola outbreak (see Table 1 for demographic information). A combination of purposive and snowball sampling was employed to recruit participants, with recruitment flyers shared via professional groups’ social media portals.^25^ Participants were recruited via phone calls, and interviews were conducted during their break hours at the hospital. All recruitment and interview activities were completed between January 2022 and June 2022.

**TABLE 1:** Participant demographic information.

Variable	*n*	%
**Work location**		
Freetown	9	37.5
Kenema	8	33.3
Makeni	7	29.2
**Gender**		
Female	17	70.8
Male	7	29.2
**Age range (years)**		
18–25	1	4.2
26–32	5	20.8
33–39	15	62.5
40–50	2	8.3
50+	1	4.2
**Marital status**		
Single (never married)	9	37.5
Married	12	50
Widow	2	8.3
Single (divorced)	1	4.2
**Health worker designation**		
Nurse	18	75
Doctor	4	16.7
Hygienist	1	4.2
Community Health Officer	1	4.2
**Years of experience**		
1–3	3	12.5
4–6	2	8.3
7–9	12	50
> 9	7	29.2
**COVID-19 vaccination status**		
Vaccinated	21	87.5
Unvaccinated	3	12.5

*Source:* David I, Tefera GM, Majee W. Beyond the needle: A qualitative exploration of Sierra Leonean healthcare workers’ post COVID-19 vaccination experiences. Health Promot Int. 2024;39(4):daae092. https://doi.org/10.1093/heapro/daae092

COVID-19, coronavirus disease 2019.

### Data collection

Participants were briefed on the research’s aim, confidentiality and data management practices before each interview and provided verbal consent to participate. The interviews were conducted in English or Krio, depending on the participant’s preference, and lasted an average of 40 min, ranging from 24 min to 70 min. The interviews were audio recorded and transcribed verbatim or translated by the first author and professional translators in Sierra Leone.

### Measures

Semi-structured interviews were conducted using an interview guide to gather detailed descriptions of healthcare workers’ experiences during the pandemic. Examples of such interview questions include: What is your impression of COVID-19? What do you think about your risk of getting COVID-19? What are your thoughts on the recommended COVID-19 prevention practices? In your opinion, how do society members view illness from COVID-19? These interview prompts were used to start the discussions with relevant follow-up prompts provided based on the participants’ responses. Responses from these conversations were used to assess perceptions of COVID-19.

### Data analysis

Thematic analysis was utilised to inductively identify themes and patterns in the overall data.^26,27,28,29^ A coding scheme was established from five randomly selected transcripts and used to code the remaining transcripts, utilising Atlas.ti qualitative analysis software.^30^ To ensure rigour in the thematic analysis, emerging themes were reviewed, defined and added to the codebook.^31,32^

For this study, a specific round of review of the data was performed to define themes, validate the codebook, deductively code based on the constructs of the HBM and TPB and analyse the themes iteratively. The HBM and the TPB were, therefore, employed as analytical lenses to understand healthcare workers’ COVID-19 prevention practices. These frameworks provided a structured approach to interpreting the data, helping to identify key factors influencing COVID-19 prevention behaviour. The data were summarised and grouped into themes, with particular attention given to the experiences of Sierra Leonean healthcare workers and how these experiences aligned with the constructs of the HBM and TPB. Pseudonym initials are used in the results section in place of the participants’ real names.

### Researcher positionality and reflexivity

All in-depth interviews were conducted by the first author, a Sierra Leonean physician who used to practice medicine in Sierra Leone but is now based at a US research university. The first author also held clinical and disease surveillance roles during the 2014 Ebola outbreak. The first author’s background encouraged participants to share their experiences openly, enhancing data quality. Additionally, other authors assisted in interpreting the data and reviewing themes, which helped minimise potential bias and improve trustworthiness.

### Ethical considerations

Ethical clearance to conduct this study was obtained from the University of Missouri-Columbia Institutional Review Board (No. 354199) and the Sierra Leone Ethics and Scientific Review Committee (SLESRC). Informed consent was obtained from all individual participants included in the study.

## Results

**FIGURE 1:**
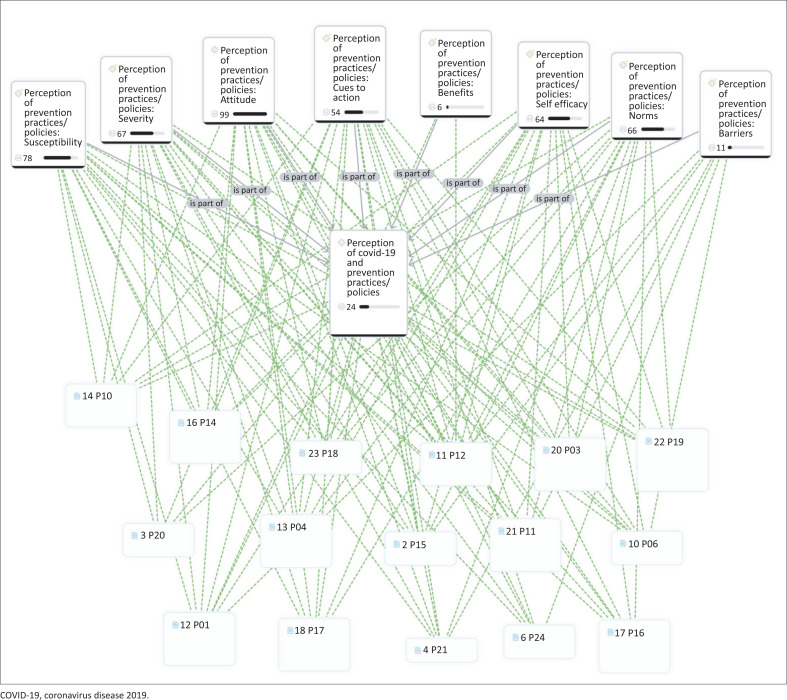
Code density chart.

The study included 24 healthcare workers (see Table 1), with a majority identifying as female (70.8%). Most participants were aged between 33 years and 39 years (62.5%), half were married (50%) and nurses comprised the predominant professional designation (75%). Participants were distributed across three key locations: Freetown (37.5%), Kenema (33.3%) and Makeni (29.2%). Most had 7–9 years of experience (50%), and a significant majority were vaccinated against COVID-19 (87.5%). Guided by the constructs of the HBM and the TPB, the main themes that emerged from participant narratives highlighted the role of attitudes and perceptions towards COVID-19 in shaping prevention practices among healthcare workers in Sierra Leone (see Table 2 for summary of themes and Figure 1 for a code density chart). Figure 1 illustrates the relationships between the parent code, its child codes and participant identifications (IDs). Quotation mark icons indicate code density for each code. Participant IDs and document numbers depict how responses are represented within the codes, highlighting their alignment and thematic patterns. Perceptions of susceptibility, severity and the benefits of COVID-19 prevention practices influenced positive health behaviours, while perceived barriers and negative subjective norms impeded such practices.

**TABLE 2:** Summary of themes.

Focus area	Themes	Sub-themes
COVID-19 prevention behaviour	1. Health workers’ risk perception and beliefs	1.1 Perceived severity and susceptibility 1.2 Influence of the Ebola outbreak 1.3 Attitudes and perceived benefits of preventive measures 1.4 Self-efficacy and perceived behavioural control
	2. Adherence to preventive measures	2.1 Facilitators: Social norms and cues to action 2.2 Barriers: Supply shortages 2.3 Barriers: Social disapproval and stigma

COVID-19, coronavirus disease 2019.

### Theme 1: Healthcare workers’ risk perceptions and beliefs

Participants’ discussions of attitudes, self-efficacy and perceptions of infection prevention practices were frequently influenced by their experiences with the Ebola outbreak, as they initially perceived COVID-19 to be equally lethal.

#### Sub-Theme 1.1: Perceived severity and susceptibility

At the time of the in-depth interviews, participants expressed varying levels of perceived risk to COVID-19 infection. Some believed their healthcare roles put them at increased risk, as described by one participant:

‘Because I am a healthcare worker, my chances of infection with COVID-19 are high, especially considering my interaction with different patients on a daily basis. In most cases, we only get to know that they are positive for COVID-19 after interactions with them. So, I think my chance of getting COVID-19 is high.’ (C, female, nurse)

However, as the COVID-19 outbreak continued and rates of new infections decreased, perceptions of risk diminished. Other participants expressed a low perception of risk based on the declining rates of COVID-19 infection in the country. When asked about his chances of getting infected with COVID-19, one participant said:

‘Presently my chances of COVID-19 infection are low compared to what it was before. This is because we haven’t recorded cases in a very long time. Initially, my chances were high because we didn’t even know who was infected with COVID-19 and who was not, but we knew that we had COVID-19 in our communities.’ (AL, male, doctor)

Eventually, healthcare workers observed that COVID-19 was not as lethal as initially feared. This change in perception of severity was influenced by their real-world experiences with the disease, which often presented with milder symptoms than anticipated. A medical doctor shared his thoughts as follows:

‘We witnessed mild to moderate COVID cases and seldomly critical cases. For instance, here in my assigned district, we had over 120 admissions for COVID-19 and only had three deaths. Our death cases were mostly people who came down with severe COVID-19 illness due to other underlying health conditions. We had up to four waves of COVID-19 community transmission, and the impact was similar.’ (J, male, doctor)

Participants’ narratives illustrate the dynamic nature of perceived susceptibility during the COVID-19 pandemic and how real-world experiences moderated their initial fears.

#### Sub-Theme 1.2: Influence of the Ebola outbreak

Further discussions with participants revealed that their perceptions of COVID-19 risk and severity were significantly influenced by their experiences with Ebola. The severe impact of the Ebola outbreak shaped healthcare workers’ initial perceptions of COVID-19, leading to heightened fear. A nurse shared her experiences as follows:

‘When we had our first COVID-19 cases, it was like COVID and Ebola are almost the same, but COVID-19 was even thought to be worse because as soon as you breathe in the virus, you’re infected.’ (P, female, nurse)

It did not take long for healthcare workers to realise that COVID-19 was not as severe as they had initially feared, having assumed that its severity would be similar to that of Ebola. Therefore, participants’ impressions of COVID-19 severity were constantly shaped by their experiences with Ebola. One participant shared her thoughts, making comparisons between COVID-19 and Ebola and explaining why she was less fearful of COVID-19:

‘I believe that COVID is real, it’s just that it did not affect us as hard as the Ebola virus which destroyed many lives in Sierra Leone. COVID is better for us when compared to the European countries, as these countries suffered greatly from COVID-19 than Sierra Leone. In Sierra Leone, we suffered greatly from the Ebola virus. So, there is a great difference. With COVID as long you take precautions and follow the COVID-19 protocols [*using your mask and avoiding public areas*], you will be fine.’ (C, female, nurse)

#### Sub-Theme 1.3: Attitudes and perceived benefits of preventive measures

Healthcare workers recognised the benefits of preventive measures such as masking and handwashing. These practices were deemed essential for preventing infections, both during and beyond the COVID-19 pandemic. One participant shared his thoughts as follows:

‘I like using face masks and regular hand washing. Those two are very important even without COVID. I’m glad that when COVID came we put more emphasis on these practices, but we have many other disease conditions that are transmitted airborne, and with most people having the habit of touching surfaces and using their fingers to touch parts of their eyes and mouth mucosa, people could easily infect themselves with other diseases.’ (AL, male, doctor)

Participants’ attitudes towards COVID-19 prevention behaviours were generally positive, with face masks and hand washing deemed essential practices. The complementary nature of these prevention measures was highlighted by a nurse in her comments:

‘I like masking and frequent handwashing the most. Because those two are very important. You could wear a mask but touch infected surfaces. We all have a tendency to touch our eyes and mouth and could easily infect ourselves.’ (CH, female, nurse)

Protection and safety were seen as major benefits of adhering to COVID-19 prevention practices. A nurse shared her thoughts as follows:

‘I like frequent hand washing and facemask even as I am talking to you now, I have my facemask. I use the mask all the time. Even when I am not working … I like all of them [*referring to COVID-19 prevention practices*] because they help keep us safe.’ (I, female, nurse)

Participants generally expressed a positive attitude towards COVID-19 prevention practices, likely influenced by the perceived safety benefits. This sub-theme reflects key constructs of both the HBM and the TPB in describing healthcare workers’ practices during the COVID-19 pandemic.

#### Sub-Theme 1.4: Self-efficacy and perceived behavioural control

The study participants reported high self-efficacy, which they believed enhanced their consistent adherence to preventive measures. Their confidence in their ability to maintain these practices was evident, even under challenging conditions, as noted by a nurse:

‘I always keep the mask on. I’ve had many colleagues ask me if I don’t get tired of the face mask and I always tell them that I’m working in an isolation unit and if I’m not using face mask people will be uncomfortable near me.’ (AB, male, nurse)

Another participant expressed her commitment to COVID-19 prevention practices, particularly masking, despite the logistical constraints experienced in her unit:

‘We are implementing all the recommended preventive measures, though it’s often challenging because there are no gloves in the wards and sometimes we don’t have access to face masks.’ (DO, female, nurse)

The study participants demonstrated confidence in their ability to maintain COVID-19 prevention practices under varying circumstances, reflecting the self-efficacy and perceived behavioural control constructs of the HBM and the TPB, respectively. Understanding healthcare workers’ perceptions, attitudes and self-efficacy towards COVID-19 prevention is also essential to examining their adherence to preventive measures, which are often motivated by these factors.

### Theme 2: Adherence to preventive measures

Social norms and cues to action, such as peer influence and institutional policies, played a significant role in maintaining prevention behaviours among healthcare workers. Participants expressed a positive perception of COVID-19 prevention practices, but adherence to these practices was influenced by various factors. Facilitators included positive social norms and cues to action, while barriers involved logistical constraints and negative social norms.

#### Sub-Theme 2.1: Facilitators – Positive social norms and cues to action

Positive social norms around COVID-19 prevention at workplaces, homes, communities and public spaces provided much-needed support for maintaining these practices. Such norms were reported to increase the perception of safety and validate healthcare workers’ efforts in preventing the spread of COVID-19. One participant expressed how these norms at their religious facility enhanced feelings of safety and motivated them to continue adhering to COVID-19 prevention practices:

‘I’m a Catholic, and since COVID started social distancing has been a priority. Seats meant for 6 people are being occupied by three people now, there are bold inscriptions on where to not sit. They have a hand washing station and all. This has been crucial in making me feel safe and keeps me motivated to lead by example.’ (A, male, nurse)

Another participant expressed his satisfaction with the mandatory masking policies implemented in the region, as they help keep everyone safe and make it easier to adhere to COVID-19 prevention practices:

‘I was happy that the policy was implemented for mandatory masking and hand washing because it keeps everyone doing safe practices.’ (AL, male, doctor)

Having adequate supplies of PPE was a key motivator for continued adherence and served as a cue to action for recommended COVID-19 prevention practices. Consequently, participants whose principal roles were at COVID-19 isolation and treatment units benefited from such cues, as PPE was available and regular compliance checks were conducted:

‘Working during the COVID-19 pandemic presented its challenges, yet it was manageable due to the comprehensive protective gear provided, covering us from head to toe. The doctors in charge ensured we had ample supplies, significantly reducing our stress during this period. However, the experience contrasted starkly with the difficulties we faced during the Ebola outbreak.’ (MA, female, hygienist)

The participant also continued by acknowledging the role of compliance checks within their unit in ensuring that they keep up with these recommended practices:

‘[*W*]e were also checked to see that we are properly protected before entering the COVID-19 unit. We had soap, water, chlorine and Dettol.’ (MA, female, hygienist)

Social norms and cues to action were therefore essential aspects of adherence to COVID-19 prevention practices.

#### Sub-Theme 2.2: Barriers – Supply shortages

Supply shortages were perceived as significant barriers for healthcare workers to maintain consistent mask use. These challenges were exacerbated by the temperate conditions within health facilities, making adherence to masking practices extremely difficult. Such frequent lack of adequate infection prevention and control (IPC) materials, including masks, meant healthcare workers often had to wear a single mask throughout an 8-h shift. As the mask accumulated sweat and became wet, it grew increasingly uncomfortable to wear:

‘It has not been easy, mostly because wearing a mask continuously gets difficult. It’s usually hot and sometimes we wear these masks until they’re wet with sweat and there isn’t sufficient mask at the hospital … We usually seek to change the mask after 4 hours. However, because of mask shortages, it’s not feasible on most days. Sometimes, we bring our masks from home and don’t have another to spare so we have to keep it on regardless … Hence, we haven’t got an option to change the wet masks. Many times, we’re buying masks ourselves to get to work because there are shortages all the time. In order to protect ourselves, we just have to buy a mask. We also have to set good examples for the patients.’ (R, female, nurse)

#### Sub-Theme 2.3: Barriers – Social disapproval and stigma

Healthcare workers also reported facing disapproval and stigma from within their social circles, making adherence to preventive measures more challenging. This social pressure often undermined their efforts to protect themselves and others. Consequently, the continued practice of COVID-19 prevention recommendations was often done with limited support from friends and sometimes even loved ones:

‘Some individuals in my social circle and in the community will say COVID-19 is not something serious, and there are people who don’t even want to hear about COVID-19. As you start talking about COVID-19, or they see you wearing a face mask, they turn their back on you and just leave.’ (AB, male, nurse)

The difference in public perception between Ebola and COVID-19, where Ebola’s symptoms were more visible, contributed to scepticism and stigma from members of their social circles regarding COVID-19 prevention practices:

‘Because with Ebola, the signs and symptoms were visible, unlike COVID-19 where you can be healthy and strong, but yet infected with COVID-19. The layperson will not believe such ideas. Even some of our colleagues were in doubt; initially, they thought it was a money-making scheme by the government. My patients, colleagues, friends, and even loved ones are among this group.’ (MAR, female, nurse)

Perceived barriers and subjective norms may have increased the difficulty maintaining COVID-19 prevention practices. However, healthcare workers were reportedly likely to adhere to prevention recommendations regardless of these challenges.

## Discussion

The HBM and the TPB are valuable in understanding the complex nature of Sierra Leonean healthcare workers’ COVID-19 prevention practices. Healthcare workers’ experiences during the 2014 Ebola outbreak significantly influenced their perceptions of the severity of the COVID-19 outbreak, particularly in its early stages. This heightened sense of severity led to strong adherence to preventive measures, demonstrating the HBM constructs of perceived severity and susceptibility. These findings support previous research demonstrating the relevance of the HBM and TPB in understanding various health behaviours, including infection prevention practices among healthcare workers.^33,34,35^ However, unlike other regions that had not experienced a major disease outbreak before COVID-19, healthcare workers in Sierra Leone compared the COVID-19 infection with Ebola. This finding suggests that their perception of COVID-19 was shaped by their previous experiences with Ebola, providing a unique context for understanding their attitudes towards the pandemic.

The attitudes of healthcare workers towards COVID-19 prevention practices were predominantly favourable, with expressions of appreciation for infection prevention measures such as hand washing, masking and social distancing. These findings align with those of other low-income settings where healthcare workers have demonstrated a general endorsement of COVID-19 prevention practices.^36,37^ The study participants’ compliance and endorsement of IPC measures were driven by their perceptions of the potential benefits of these practices, aligning with the HBM constructs of perceived benefits and self-efficacy. Their familiarity with similar measures during the Ebola outbreak further reinforced their commitment, highlighting the TPB constructs of attitude and perceived behavioural control. This underscores the importance of prioritising evidence-based interventions that have been proven effective in controlling infectious diseases.

The Ebola outbreak had a significant impact in Sierra Leone, particularly on healthcare workers who lost several colleagues to Ebola infection while carrying out their duties.^24^ Consequently, COVID-19 was initially perceived as equally severe and potentially fatal, especially in low-resource settings like Sierra Leone, reflecting the HBM construct of perceived severity. Reports of severe respiratory failure and deaths among COVID-19 infected persons in high-income settings further amplified this fear.^38^ However, as healthcare workers managed COVID-19 cases and observed outcomes, their initial fear abated, especially when comparing COVID-19 outcomes with those of Ebola. Between 30 March 2020 and 22 February 2023, Sierra Leone recorded 7760 cases and 126 deaths from COVID-19, significantly lower than the Ebola outbreak’s 14 124 cases and 3956 deaths between 2014 and 2016.^39,40,41^ This shift illustrates the dynamic nature of perceived severity over time, as posited by the HBM.

The perceived susceptibility of healthcare workers was influenced by their frontline roles and potential exposure to patients whose COVID-19 status was often unknown during visits. Despite confirmation of local COVID-19 transmission, infection rates remained low, reducing perceived susceptibility over time.^42^ The study’s findings highlight how past experiences with Ebola heightened initial perceptions of COVID-19’s susceptibility severity, which then moderated as real-world outcomes were observed. These findings underscore the importance of the HBM construct of perceived susceptibility in shaping health behaviours.

Maintaining preventive measures was challenging because of barriers such as mask availability and temperature conditions in health facilities. Sierra Leone’s warm and humid climate, combined with limited air conditioning in health facilities, made mask-wearing particularly difficult.^43^ Similar barriers have been reported in other low-middle-income settings.^44,45^ Despite these challenges, healthcare workers in Sierra Leone prioritised and adhered to preventive practices, likely driven by their fear of potential infection. This behaviour illustrates the interplay between the HBM constructs of perceived barriers and susceptibility and the TPB construct of perceived behavioural control.^46,47^

Healthcare workers’ efforts to practise COVID-19 prevention measures were motivated by the availability of PPE but hindered by scarcity and disapproval from the general population, which also held conspiracy theories about COVID-19. This lack of support made adherence to prevention practices more challenging. Existing literature suggests that stigma or disapproval from society can deter maintaining COVID-19 prevention practices, aligning with the TPB construct of subjective norms.^48,49^ The findings suggest that the HBM and TPB constructs are complementary, as evidenced by the participants’ experiences.

### Study limitations and implications for future research

The in-depth interviews predominantly took place during the participants’ break periods at their workplaces, which occasionally resulted in shortened discussions. A more extended engagement might have allowed some participants to delve deeper into their experiences with COVID-19 prevention practices. Nonetheless, the interviewer’s profound understanding of the local culture and healthcare system, combined with their extensive experiences in qualitative research methods, facilitated substantive conversations within the constraints of the participants’ available time.

Future research should focus on exploring prevention practices among the general population in Sierra Leone to see if these findings are similar among individuals with limited health information and education. Additionally, future research initiatives should strive to compare these findings with those of other healthcare workers in the West African region who share similar cultures with Sierra Leone but without similar experiences from the Ebola outbreak. In striving for global recovery from the COVID-19 pandemic, public health preparedness efforts must incorporate lessons from the crisis to enhance IPC in low-resource settings.

## Conclusion

The study findings emphasise the role of context in shaping COVID-19 prevention behaviour. As predicted by the HBM and the theory of planned behaviour, the findings highlighted several factors that influence healthcare workers’ adherence to preventive measures. Health interventions aimed at improving COVID-19 prevention behaviour among healthcare workers in Sierra Leone should focus on addressing the identified barriers to adherence, such as improving access to PPE and providing more education and awareness-raising activities about the importance of IPC measures. Furthermore, efforts to combat disapproval and conspiracy theories should be intensified through community engagement programmes that involve healthcare workers and community leaders. It is also recommended that national policies and interventions prioritise evidence-based interventions that have been proven effective in controlling infectious diseases, such as handwashing, masking and social distancing, over restrictions that may have adverse economic impacts on the general population. The findings of this study have important implications for improving infectious disease prevention practices among healthcare workers in Sierra Leone. Strengthening these practices could be crucial for controlling the spread of COVID-19 and enhancing preparedness for future public health emergencies.
